# IL-21 Optimizes the CAR-T Cell Preparation Through Improving Lentivirus Mediated Transfection Efficiency of T Cells and Enhancing CAR-T Cell Cytotoxic Activities

**DOI:** 10.3389/fmolb.2021.675179

**Published:** 2021-06-04

**Authors:** Li Du, Yaru Nai, Meiying Shen, Tingting Li, Jingjing Huang, Xiaojian Han, Wang Wang, Da Pang, Aishun Jin

**Affiliations:** ^1^ Chongqing Key Laboratory of Basic and Translational Research of Tumor Immunology, Chongqing Medical University, Chongqing, China; ^2^ Department of Breast Surgery, Harbin Medical University Cancer Hospital, Harbin, China

**Keywords:** CAR-T, IL-21, transfection, IFN-γ, cytotoxicity

## Abstract

Adoptive immunotherapy using CAR-T cells is a promising curative treatment strategy for hematological malignancies. Current manufacture of clinical-grade CAR-T cells based on lentiviral/retrovirus transfection of T cells followed by anti-CD3/CD28 activation supplemented with IL-2 has been associated with low transfection efficiency and usually based on the use of terminally differentiated effector T cells. Thus, improving the quality and the quantity of CAR-T cells are essential for optimizing the CAR-T cell preparation. In our study, we focus on the role of IL-21 in the γ_c_ cytokine conditions for CAR-T cell preparation. We found for the first time that the addition of IL-21 in the CAR-T preparation improved T cell transfection efficiency through the reduction of IFN-γ expression 24–48 h after T cell activation. We also confirmed that IL-21 enhanced the enrichment and expansion of less differentiated CAR-T cells. Finally, we validated that IL-21 improved the CAR-T cell cytotoxicity, which was related to increased secretion of effector cytokines. Together, these findings can be used to optimize the CAR-T cell preparation.

## Introduction

CAR-T cell therapy has shown great promise in the advancement of individualized cancer immunotherapy (Maude et al., 2018; Park et al., 2018). However, there are still some formidable challenges for broader application of the CAR-engineered T cells, including the low transfection efficacy of T cells, suppression of CAR-T cell function, and the lack of expansion and persistence of CAR-T cells after infusion (Junttila and de Sauvage, 2013; Joyce and Fearon, 2015; O'Rourke et al., 2017). Thus, it is of great importance to optimize the production methods to obtain CAR-T cells with advanced quality and potentials for antitumor immunotherapy.

Lentivirus vectors are capable of stably infecting the dividing or non-dividing cells by integrating into the host genome (Bukrinsky et al., 1993; Schambach et al., 2013). Moreover, these vectors are nontoxic to the human body since no viral genes are integrated into the vector genome which enables transduced T cells to obtain long term stable gene expression (Knight et al., 2012). Thus, lentiviral transfection has gained full attention in immunotherapy research. But a limitation of lentivirus-mediated T-cell transfection is the low transfection efficacy (Kennedy and Cribbs, 2016), and PBMC-derived T cells from cancer patients has been reported with even less transfection efficacy comparing with T cells from healthy donors (Su et al., 2016). To date, how to increase the lentiviral transfection efficiency is still challenging.

Considering the limited persistence of CAR-T cells in patients, one promising approach to enhance the efficacy of CAR- T cells is to increase the percentages of less differentiated T cell phenotypes that have superior proliferation capacity, such as naive T (Tn) and central memory T (Tcm) (Alizadeh et al., 2019). Despite a clear advantage of the less-differentiated populations, the majority of ACT trials utilize unfractionated T cell subsets (Sabatino et al., 2016; Jackson et al., 2020), due to the lack of appropriate methods for generation of less differentiated CAR-T cells.

The common γ_c_ family consists of six cytokines. Among them, IL-2, IL-7, IL-15 and IL-21 have been widely utilized for the CAR-T cell preparation, because these cytokines play pivotal roles in fueling T cells to thrive, combat tumors and drive long-lived memory against tumor metastasis or relapse (Hashimoto et al., 2019; Shourian et al., 2019; Dwyer et al., 2019). Even though the γ_c_ family bind to and signal through the common γ_c_ receptor to support T cell proliferation and differentiation, each cytokine has a distinctive role in T cell growth, lineage control/determination, differentiation, and function (Cui et al., 2011; Adachi et al., 2018; Lanitis et al., 2021). IL-21 has been reported to predominantly activate signal transducer and activator of transcription 1 (STAT1) and STAT3 and, to a lesser degree, STAT5A and STAT5B, but IL-2, IL-7 and IL-15 preferentially activate STAT5A and STAT5B (Leonard and Spolski, 2005; Zeng et al., 2007). Due to this difference, IL-21 has its own unique roles in anti-tumor immunity. Although it has been reported that IL-21 alone has minimal effects on CD8^+^ T cell proliferation (Zeng et al., 2007), IL-21 functions synergistically with other cytokines to promote T cell proliferation and memory T cell formation, and enhance the antitumor efficacy of effector cells (Markley and Sadelain, 2010).

In the present study, we found that IL-21 improved the lentiviral transfection efficiency of T cells through decreasing the IFN-γ expression before T cell transfection. Further, IL-21 increased the proportions of less differentiated CAR-T cells and further enhanced their proliferative capacity. Lastly, IL-21 effectively augmented the cytotoxicity of human epidermal growth factor receptor-2 (HER-2) CAR-T cells against HER-2-positive cancer cells. Collectively, these findings evidenced the benefits of IL-21 in the CAR-T cell preparation prior to their use in adoptive T-cell therapy, and provided a potential explanation for its role in improving the transfection efficiency of T cells.

## Materials and Methods

### Cells and Culture Conditions

SK-BR-3 and HS578T cell lines were obtained from ATCC and cultured in Dulbecco’s modified Eagle medium (Gibco, Invitrogen, Carlsbad, CA) supplemented with 10% fetal bovine serum (FBS) (Biological Industries, ISR). SW480 was obtained from ATCC and cultured in Roswell Park Memorial Institute (RPMI) 1640 Medium (Gibco, Invitrogen, Carlsbad, CA) supplemented with 10% fetal bovine serum (FBS) (Biological Industries, ISR). HEK293T cell line (ATCC) was maintained in complete DMEM (Gibco, Invitrogen, Carlsbad, CA) supplemented with 10% FBS (Gibco, Invitrogen, Carlsbad, CA), 4 mM L-glutamine (Gibco, Invitrogen, Carlsbad, CA) and 1 mM sodium pyruvate solution (Gibco, Invitrogen, Carlsbad, CA). These cells cultured under an atmosphere containing 5% carbon dioxide were routinely tested to exclude infection with Myoplasma.

### Plasmid Construction and Lentivirus Production

**FIGURE 1:**
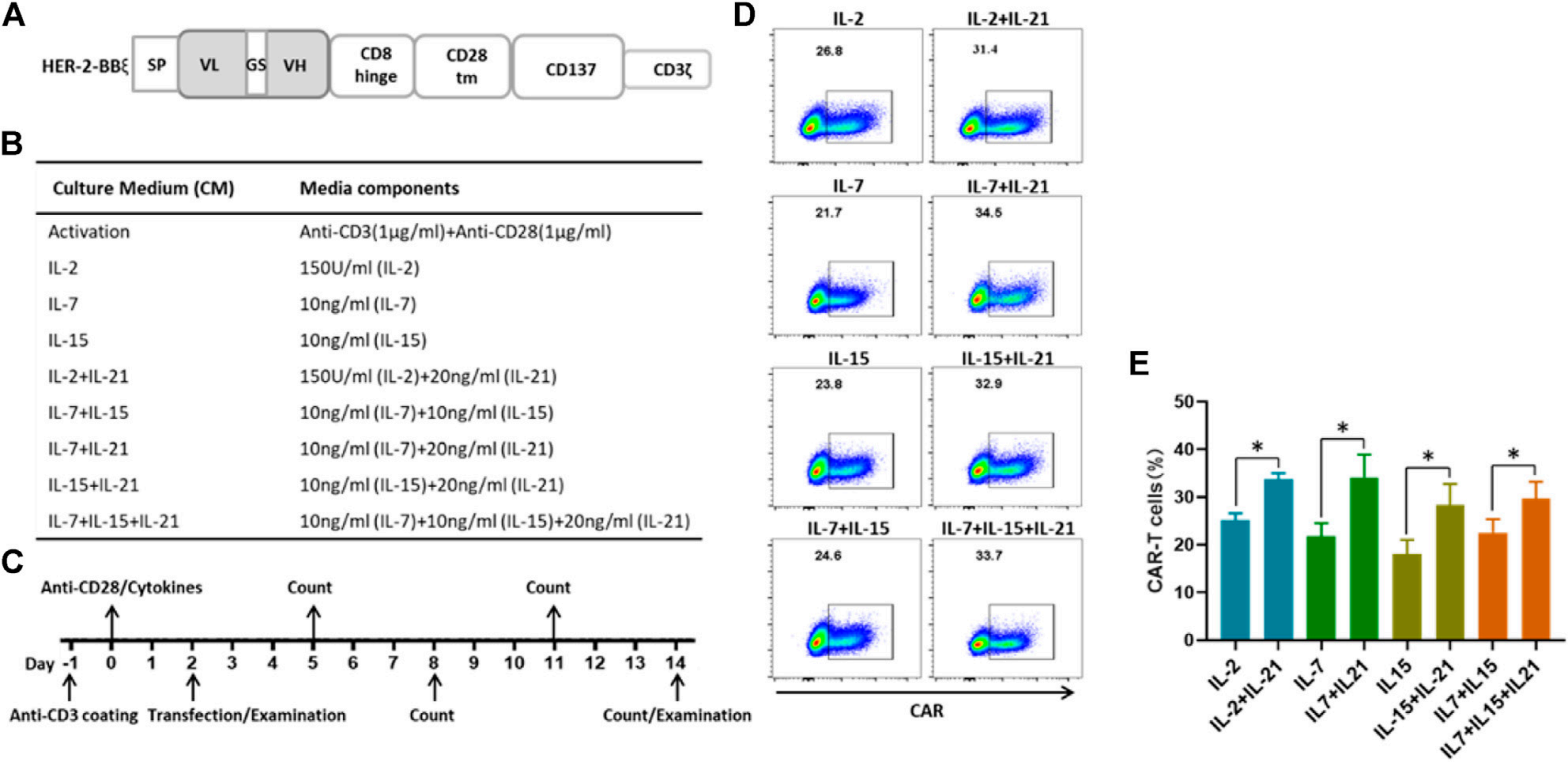
IL-21 enhances the transduction efficience of T cells **(A)** Schematic diagrams of lentiviral HER-2 CAR structures. **(B)** Culture conditions of different protocols for the generation of HER-2 CAR-T cells. **(C)** Time schema of main experimental manipulation for the generation of HER-2 CAR-T cells. **(D,E)** CAR expression in CD3^+^T cells transfected using lentiviral vectors containing the indicated HER-2-CAR constructs as measured by flow cytometry on the 12th day after transfection. Data from one representative donor and summary for three independent donors in independent expansions was shown (mean ± SEM), results were compared using Student’s *t*-test (*p < 0.05, **p < 0.01).

The HER-2- specific scFv sequence was derived from the humanized mAb that was used to produce Herceptin (Carter et al., 1992; Zhao et al., 2009), which was fused with the transmembrane region of CD8 (nucleotides 1,271–1,519; GenBank accession number NM_001768.6), co-stimulatory domain CD28 (nucleotides 760–882; NM_006139.3), co-stimulatory domain CD137 (nucleotides 901–1,026; NM_001561) and CD3ζ (nucleotides 299–637; NM_198053.2) by overlapping PCR. PCR products were then cloned into the PWPXL lentivirus vector, CAR structures are showed in Figure 1A. All generated plasmids were subjected to sequencing verification and used for downstream applications. To obtain lentiviruses, lentivirus was packaged and produced as previously described. In brief, 293-T packaging cells were seeded into six-well plates (8 × 10^5^ cells/well) for 24  h, then transfected with plasmids encoding CAR moieties, pMD.2 G encoding VSV-G envelope, and a packaging vector psPAX2 using Xfect Transfection Reagent (Takara) following the manufacturer’s instructions. The virus supernatant was harvested at 48 h after transfection, filtered through a 0.45 μm membrane and stored at −80°C.

### Transfection and Expansion of Human T Cells

Peripheral blood mononuclear cells (PBMCs) were obtained from healthy donors (*n* = 4) after informed consent, isolated by using MACSprep™ PBMC Isolation Kit (Miltenyi Biotec, Germany), according the manufacturer’s instructions. The PBMCs were activated for 48 h in tissue culture-treated 24-well plates (2 × 10^6^/well) with anti-CD3 mAb (Miltenyi, Biotec, plate-bound, 1 ug/ml) and anti-CD28 mAb (Miltenyi, Biotec, soluble, 1 ug/ml) in complete medium (90% RPMI-1640 supplemented with 10% FBS (Gibco), 2 mM L-glutamine, 25 mM HEPES, 55 μM 2-M,100 U/ml penicillin, 100 μg/ml streptomycin and different cytokine combinations), and various cytokine conditions as shown in Figure 1B. After 2 days, 2 × 10^5^ activated T cells were transfected with 500 μL the CAR-containing lentiviral supernatant in 24-well tissue culture-treated plates, and the supernatant was replaced with the fresh corresponding medium at 24 h after transfection. Culture medium change with fresh addition of cytokines was performed every other day. The T cells were transferred to 12-well tissue culture plates or 6-well tissue culture plates depending on the total cell number, and cell cultures were maintained at 37°C with 5% CO_2_. On the 12th day after transfection, the T cells were collected for subsequent experiments.

### Flow Cytometry

For cell membrane staining, 1 × 10^6^ cells were incubated with 2 μg/ml fluorescent antibodies at room temperature for 15 min protected from light. For intracellular staining, after cell surface staining, cells were fixed and permeabilized with Cytofix/CytoPerm (BD Biosciences) for 20 min on ice and washed with 1× PermWash (BD Biosciences). Subsequent staining was performed in the same manner described above with 1× PermWash as the staining and wash buffer. The following antibodies used for the flow cytometry analysis were obtained from Biolegend, United States: FITC-conjugated anti-CD3 (clone SK7), BV510-conjugated anti-CD3 (clone SK7), Percy5.5-conjugated anti-CD4 (clone RPA-T4), Alex700-conjugated anti-CD8 (clone HIT8a), BV421-conjugated anti-CCR7 (clone G043H7), APC-conjugated anti-CD45RA (clone HI100), BV421-conjugated anti-PD-1 (clone EH12.2H7), BV605-conjugated anti-LAG-3 (clone 11C3C65), BV785-conjugated anti-TIM-3 (clone F38-2E2), PE-conjugated anti-IFN- (clone 4S.B3), APC-conjugated anti-human HER-2 (clone 24D2). FITC-conjugated Her-2 recombinant protein was purchased from ACRO Biosystems. All flow samples were analyzed on a BD celesta (BD Biosciences), and data was analyzed with FlowJo (version 10).

### Cytotoxicity Assay

The ability of CAR-T cells to kill tumor target cells was measured by Calcein AM (CAM, Dojindo) release-based cytotoxic cell assay (Jonsson et al., 1996). Briefly, target cells were centrifuged and resuspended with fresh culture medium in the centrifuge tubes. 2 × 10^6^ target cells were incubated with 10 µM CAM in culture medium for 30 min at 37 C protected from light. Then the culture medium containing CAM was removed and the cell pellets were washed five times with Dulbecco’s phosphate buffered saline without calcium or magnesium (DPBS). The target cell density was adjusted to 1 × 10^5^/ml with culture medium. Non-transduced T cells were used to normalize the percentage of CAR-positive cells. The CAR-T cells were adjusted to 1 × 10^6^ cells/ml with T cell culture medium, and seeded with 100 μL volume per well into 96-well plates (V bottom). The CAR-T cells in 96 well plates were diluted in gradients to the appropriate concentrations (1 × 10^6^ cells/ml, 5 × 10^5^ cells/ml and 2.5 × 10^5^ cells/ml). Then 100 μL medium containing 1 × 10^4^ target cells were added into the 96-well plates. The CAR-T cells were co-cultured with labeled target cells at different ratios (from 10:1 to 2.5:1) for 6 h, and then 50 µL of the culture supernatant was transferred to a 96-well black culture plate to measure fluorescence intensity (FI) at 485 nm excitation and 530 nm emission wavelengths. Fluorescence value of wells containing target cells alone were detected and subtracted as the background from the values of the co-cultures. Wells containing target cells alone were mixed with 1% Triton-X 100 for 6 h at 37 C and the resulting fluorescence was set as 100% lysis. Cytotoxicity was calculated according to the following formula: %Cytotoxicity = (Experimental − Target spontaneous)/(Target maximum − Target spontaneous) × 100%.

### Cytokine Measurements

For the detection of cytokine secretion by T cells, the culture supernatants of T cells were harvested at 24 h after co-culture with target cells and at different time points after activated with anti-CD3/anti-CD28. The supernatant was added into 96-well plates coated with human IFN-γ capture antibody (Clone MD-1, 2 μg/mL, Biolegend) or human Granzyme-B capture antibody (Clone GB10, 2 μg/mL, Mabtech). The plates were developed with bio-human IFN-γ detection antibody (Clone 3D1D12, 1 μg/mL, Biolegend) or bio-human Granzyme-B detection antibody (Clone GB11, 1 μg/mL Mabtech), followed by incubation with Streptavidin-ALP (1:1,000, Mabtech, Sweden) and pNPP. The plates were analyzed by the Varioskan LUX Multimode Microplate Reader for the OD405 value.

### Statistical Analysis

Statistical computations were done using the Graph Pad Prism 8.0 software. Data was presented as mean ± SEM. Statistical differences were evaluated using an unpaired Students *t*-test, and statistical significance was set at *p* < 0.05.

## Results

### The Addition of IL-21 Efficiently Increases the Transfection Efficiency of T Cells During the CAR-T Cell Preparation

Given the significance of the transfection efficiency for the CAR-T cell preparation, we compared the populations of HER-2-expressing T cells under different cytokine conditions. T cells were activated with one of the following cytokine cocktails: IL-2, IL-7, IL-15 individually, a combination of IL-7 and IL-15, or each of these four groups with the addition of IL-21, as shown in Figure 1B. Two days after T cell activation, the T cells were infected with lentivirus encoding the HER-2 CAR construct, which had been validated in our laboratory. T cell transfection efficiency was detected at day 12 post infection (Figure 1C). We found that the frequency of CAR expression ranged from 15 to 30% for T cells cultured in the commonly used cytokine conditions, and from 25 to 40% with the addition of IL-21 (Figures 1D,E). These results showed that the addition of IL-21 in the cytokine conditions was beneficial for the transfection efficiency of T cells.

### IL-21 Improves the Proliferation Capacity of Activated T Cells

**FIGURE 2:**
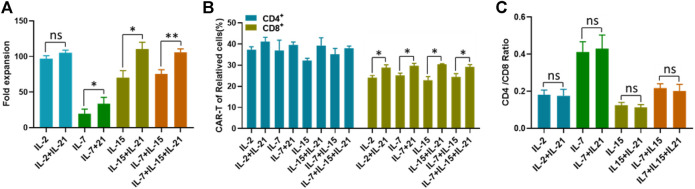
IL-21 enhances the proliferation of T cells. **(A)** The number of T cells in different culture conditions was counted with in automatic cell counter on the 12th day after transfection. The fold expansion was calculated as total number of proliferating cells/the starting cell number. **(B)** The transfection efficiency of CD4^+^T cells and CD8^+^ cells was calculated on the 12th day after transduction. **(C)** CD4/CD8 ratio of CD3^+^T cells was calculated on day 12 after T cell transfection. Data representative of three separate experiments are shown (mean ± SEM). Statistical significance was tested using Student’s *t*-test (*p < 0.05, **p < 0.01).

An important consideration of the CAR-T cell preparation is whether T cells have robust proliferative capacity. Another consideration is whether IL-21 impairs the expansion ability of T cells, and which may in turn contribute to the increased transfection efficiency of T cells. We analyzed the proliferation capacity of T cells cultured with the different cytokine conditions described. As shown in Figure 2A, the addition of IL-21 to IL-7, IL-15 or IL-7+IL-15 combo markedly enhanced T cell expansion. While no obvious differences in the proliferation of T cells were observed between IL-2 and IL-2+IL-21. Despite the slight increase of T cell numbers induced by the addition of IL-21, IL-7 was insufficient to generate desired quantity of T cells for further use. We also analyzed the expansion ability of T cells after incubation for different time periods. The results showed that the proliferative capacity of T cells decreased by degrees with time of culture. The proliferation capacity of T cells cultured under the IL-7 or IL-7+IL-21 seemed comparable to T cells cultured under the other cytokine conditions during the first 5 days of culture. While, the IL-7 and IL-7+IL-21 combos showed lower proliferation ability of T cells after 5 days of culture, compared to the other conditions (Supplementary Figure S1).

Due to the difference in transfection efficiency between CD4^+^ T cells and CD8^+^ cells reported in the literature (Brown et al., 2018), we compared the transfection efficiency between these two groups. We confirmed that CD4^+^ T cells exhibited higher transfection efficiency than CD8^+^ T cells, regardless of the cytokine conditions used (Figure 2B). However, only CD8^+^ T cells were found to be associated with the IL-21 induced transfection efficiency improvement, indicated by the significant increases of CAR-T cell proportions with the addition of IL-21 to each of the commonly used cytokine condition (Figure 2B). In order to determine whether the increased transfection efficiency of CD4^+^ T cells with lead to a dominance of these cells during expansion, we compared the CD4/CD8 ratios at the beginning and at the end of CAR-T cell preparation, especially between each culture condition with or without IL-21. The CD4/CD8 ratios among these groups were close to 1, and there was no difference of the CD4/CD8 ratios among all eight groups before the transfection procedure (Supplementary Figure S2). At the end of CAR-T cell preparation, The CD4/CD8 ratio was highest in IL-7 and IL-7+IL-21 and lowest in IL-15 and IL-15+IL-21, but no significant difference was observed between the cytokine conditions without the addition of IL-21 and the corresponding conditions with addition of IL-21 (Figure 2C). Meanwhile, The CD4/CD8 ratios among these groups were smaller than 0.5, suggesting that the cultivation induced by anti-CD3/CD28 in the presence of exogenous γ chain cytokines favored the expansion of CD8^+^ T cells (Han et al., 2018). Together, these results demonstrated that the improved transfection efficiency of T cells was not associated with T cell expansion and CD4/CD8 ratio.

### IL-21 Increases the Proportion of Tn in CAR-T Cells

**FIGURE 3:**
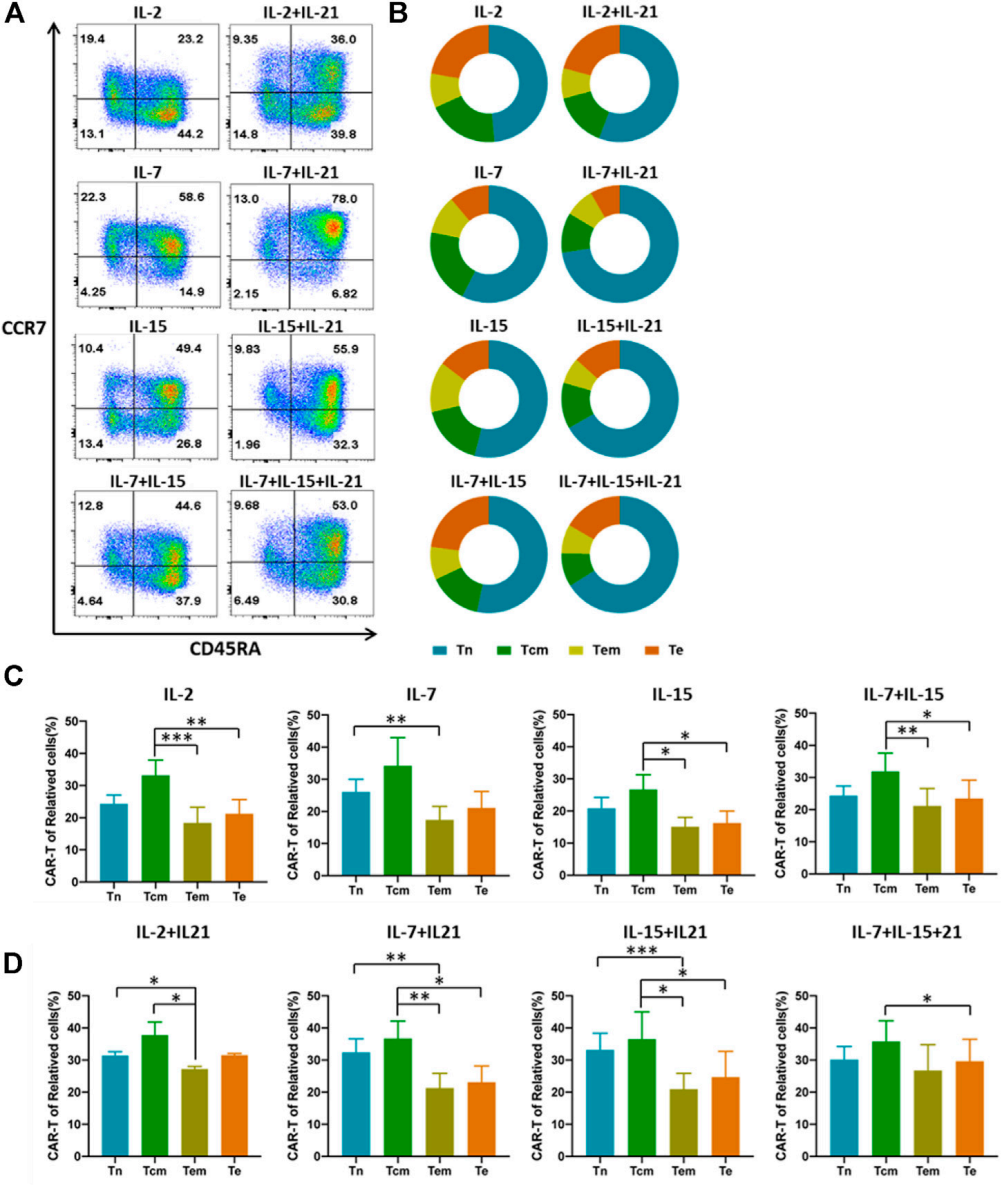
IL-21 affects the proportion of different CAR-T cell subtypes. **(A,B)** Differentiated subtypes of HER-2 CAR-T cells were measured with surface expression of CD45RA and CCR7 on day 12 after transduction by flow cytometry. Tn:CD45RA + CCR7+, Tcm:CD45RA-CCR7+, Effector memory T cells (Tem): CD45RA-CCR7−, Effector T cells (Te): CD45RA + CCR7−, Results from flow plots of one representative donor and summary data for indicated CAR-T cell groups were shown. **(C,D)** CAR expression of different cell phenotypes was shown with percentage of CAR^+^ cells out of the corresponding subtypes. Error bars represent ±SEM. Statistical significance was tested using Student’s *t*-test (*p < 0.05, **p < 0.01, ***p < 0.001).

Given the importance of IL-21 in the development and maintenance of central memory T cells by induction of an early differentiation phenotype (Loschinski et al., 2018), we assessed the differentiation of HER-2 CAR-T cells. Our data showed that CAR-T cells cultured with the addition of IL-21 had a higher proportion of T cells that exhibited a Tn phenotype as compared to the corresponding CAR-T cells cultured without IL-21 (Figures 3A,B). The IL-7+IL-21 led to the highest percentage of Tn cells, followed by IL-15+IL-21 and IL-7+IL-15+IL-21. This was consistent with the results that both IL-7 and IL-21 support the survival and homeostasis of naïve and memory T cells in the prior literature (Adachi et al., 2018). Meanwhile, IL-2 caused the generation of the lowest percentage of Tn cells, which was reported to drive terminal effector differentiation and suppress expression of makers associated with memory (Kalia et al., 2010).

It is noteworthy that whether the transfection efficiency varies among T cell subtypes. To eliminate this doubt, we analyzed the transfection efficiency in the four subtypes. We found that the transfection efficiency differed between several subtypes. Tcm phenotype had the highest transfection efficiency in most cytokine cocktails (Figures 3C,D). To explore whether the increased transfection efficiency of T cells cultured with the addition of IL-21 is associated with the differences in transfection efficiency among T cell subtypes, we compared the proportion of each T cell subtype cultured under the different cytokine conditions before the transfection procedure. The results showed that Tn accounted for the largest proportion of T cells, and no significant difference in the proportion of each T-cell subtype was observed between cytokine conditions (Supplementary Figure S3). Finally, we compared the transfection efficiency of each T cell subtype cultured with the different cytokine conditions. The data showed that the addition of IL-21 in the cytokine conditions increased the proportion of CAR-T cells in Tn. But no significant differences for CAR-T proportion in Tcm were observed across all cytokine conditions (Supplementary Figure S4). The above experiment suggested the existence of additional mechanisms for the increased transfection efficiency of T cells cultured with the addition of IL-21.

### IL-21 Regulates the Secretion of IFN-γ at the Early Stage of T Cell Activation

**FIGURE 4:**
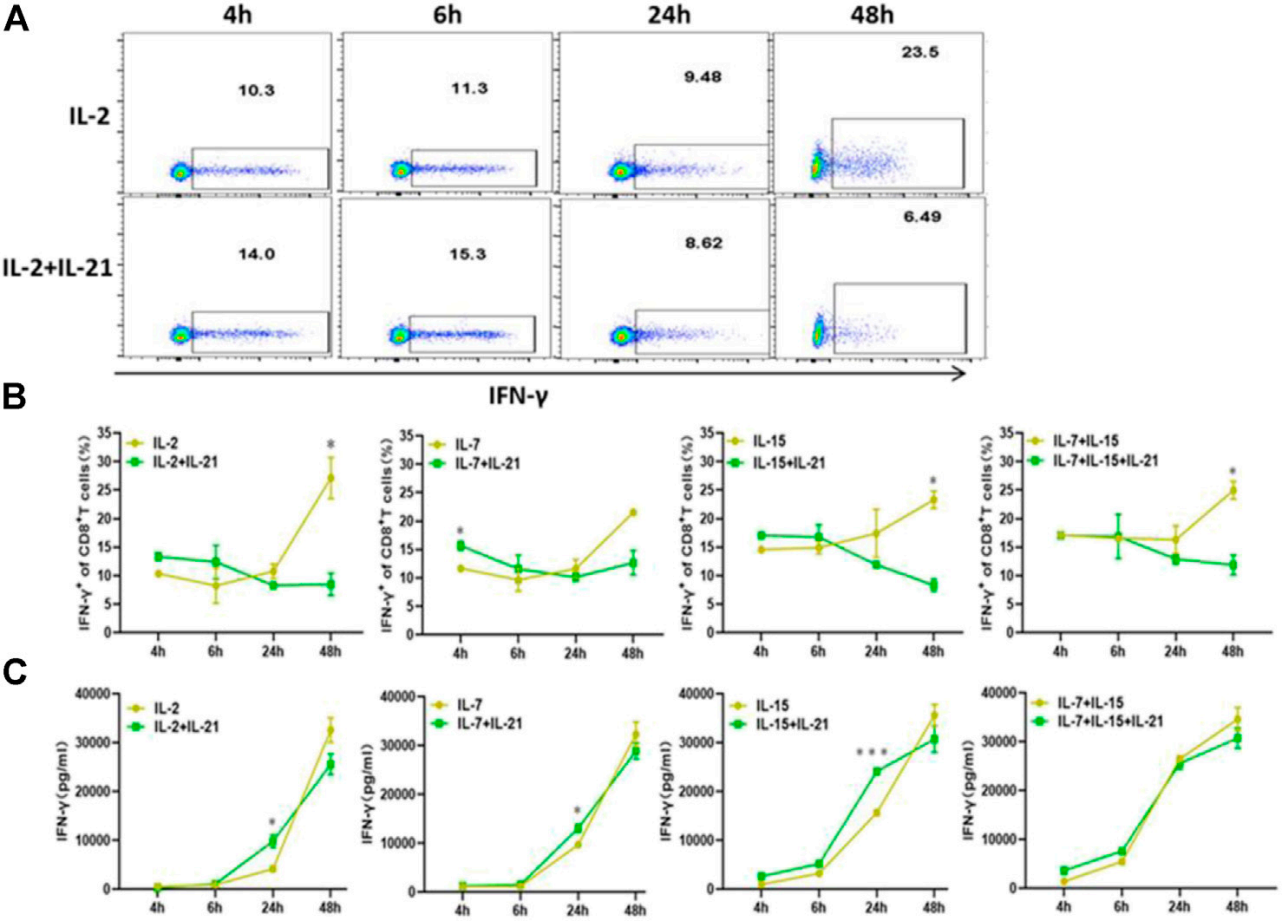
IL-21 influence the IFN-γ expression. **(A,B)** Percentages of IFN-γ^+^ T-cells among CD8^+^ T cells at different activation time points were evaluated by intracellular FACS assay. Results from flow plots of one representative donor and summary data for indicated CAR-T cell group were shown. **(C)** IFN-γ concentraction in different culture supernatants was detected by ELISA at various time points after stimulation. Each experiment was performed in triplicate, and error bars represent ± SEM. Statistical significance was tested using Students’s *T*-test (*p < 0.05, **p < 0.01, ***p < 0.001).

Previous literature had indicated that IFN-γ secretion was assessed as a measure of T cell activation and effector function, which was also known to mediate potent anti-viral immunity (Kak et al., 2018). Therefore, we examined the expression of IFN-γ at various time points. We observed that the addition of IL-21 to the IL-2, IL-7 or IL-15 combo increased the proportion of IFN-γ ^+^ T cells in the CD8^+^ T cells during the first 6 h of T cell activation. While, there was no difference in IFN-γ expression between the IL-7+IL-15 and IL-7+IL-15+IL-21 during the first 6 h of T cell activation. Unexpectedly, the proportion of IFN-γ^+^ T cells in CD8^+^ T cells cultured with the addition of IL-21 markedly decreased below that in the corresponding cytokine groups without addition of IL-21 (Figures 4A,B, Supplementary Figure S5). Since IFN-γ concentration in culture supernatants represents the results of quantification of elongated accumulation, the results of IFN-γ detection in the culture supernatant showed slight differences compared with the above results. The cytokine conditions with the addition of IL-21 exhibited higher IFN-γ secretion than the corresponding cytokine conditions without the addition of IL-21 at 24 h after T cell activation, but no obvious difference in IFN-γ secretion was observed between the IL-7+IL-15 and IL-7+IL-15+IL-21 (Figure 4C).

### The Transfection Efficiency of T Cells is Negatively Associated with Expression of IFN-**γ**

**FIGURE 5:**
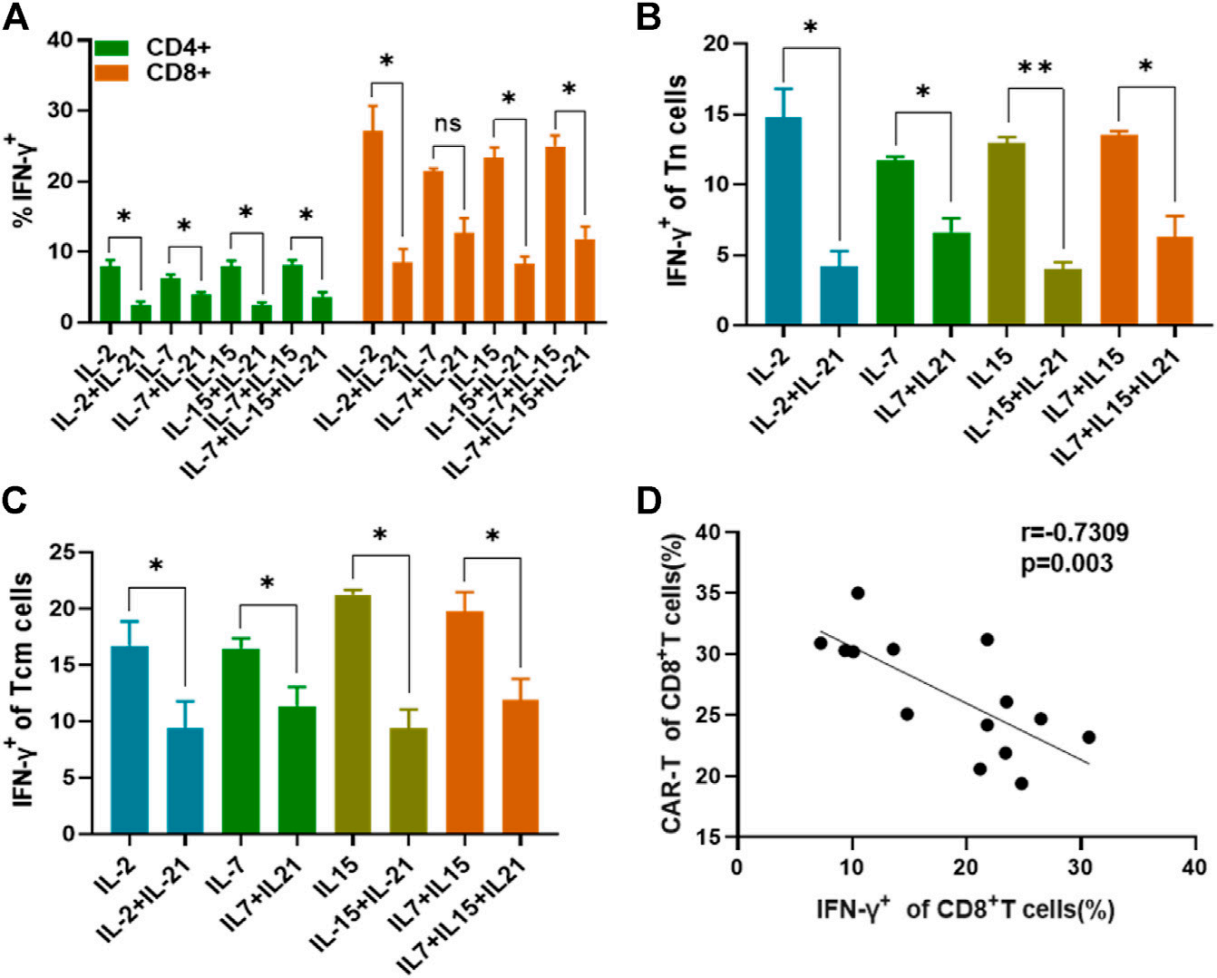
IFN-γ expression affects T cell transfection efficiency. **(A)** The IFN-γ expression of CD4^+^T cells and CD8^+^T cells among different cytokines groups was evaluated on day 2 after activation by flow cytometry. **(B,C)** The percentages of IFN-γ-producting T cells in the Tn and Tcm subtypes were analyzed at 48th after stimulation. **(D)** Analysis of the correlation between the percentage of IFN-γ^+^ CD8^+^ T cells at 48 h after stimulation and transduction efficiency of CD8^+^ T cells using a Spearman rank correlation test was shown. All experiments were performed in triplicate manner and percentage was statistically analyzed. *p < 0.05, **p < 0.01, Error bars represent ± SEM.

To further study whether the increased transfection efficiency of T cells cultured with the addition of IL-21 is associated with the IFN-γ expression, we compared the IFN-γ expression of T cells cultured under the different cytokine conditions at 48 h after activation, because transfection was performed at this moment. As shown in Figure 5A, CD8^+^ T cells exhibited a higher proportion of IFN-γ^+^ T cells than CD4^+^ T cells in each cytokine condition. Both CD4^+^ and CD8^+^ T cells cultured with the addition of IL-21 presented lower expression of IFN-γ as compared to the corresponding cytokine conditions without addition of IL-21, except for CD8^+^ T cells in the IL-7 and IL-7+IL-21. Then, we analyzed IFN-γ expression in the Tn and Tcm subtypes, due to the fact that Tn and Tcm accounted for a significant proportion of CAR-T cells. The results indicated Tn and Tcm subtypes cultured with the addition of IL-21 presented lower expression of IFN-γ than that in corresponding cytokine conditions without the addition of IL-21 (Figures 5B,C). In addition, we compared the IFN-γ expression level in the four T cell subtypes. Tem presented the highest expression of IFN-γ in the T cell subtypes (Supplementary Figure S6). Further, we analyzed the correlation between IFN-γ expression and the transfection efficiency of CD8^+^ T cells. The results showed that the transfection efficiency of CD8^+^ T cells showed significant negative correlation with its IFN-γ expression, which provided potential explanation for the increased transfection efficiency of T cells (Figure 5D).

### IL-21 Enhances the CAR-T Cell Antitumor Activity and Secretion of Effector Cytokines

**FIGURE 6:**
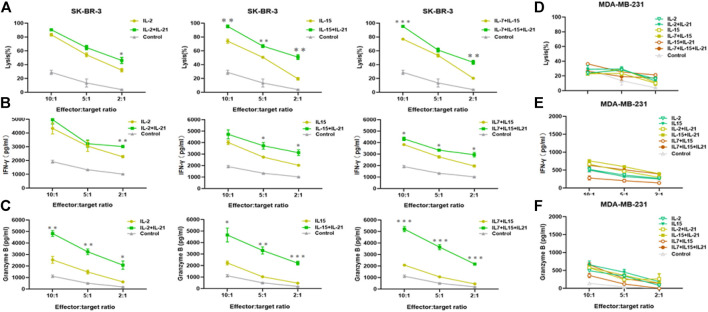
IL-21 augments cytolytic activity of HER-2-specific T cells. **(A)** Cytotoxicity of HER-2-specific CAR-T cells was evaluated by Calcein AM release assay after co-culture with HER-2-positive SK-BR-3 cells for 6 h at E:T ranging from 10:1 to 2:1. **(B,C)** Supernatant was collected after 24th co-culture of HER-2 CAR-T cells with SK-BR-3 and assayed for IFN-γ and granzyme B release by ELISA. **(D)** Cytotoxicity was evaluated by Calcein AM release assay after co-culture with HER-2-negative MDA-MB-231 cell for 6 h at E:F ranging from 10:1 to 2:1. **(E,F)** Supernatant was collected after 24 h co-culture with MDA-MB-231 cells and assayed for IFN-γ and granzyme B release. Each experiment was performed in triplicate. Mean values were calculated for each group. Stastistical was calculated using two-way Student *t* test, *p < 0.05, **p < 0.01, ***p < 0.001.

The HER-2-CAR-mediated specific tumor cell killing was explored using a Calcein AM release-based cytotoxic cell assay. First, we selected the HER-2-positive SK-BR-3 and HER-2-negative MDA-MB-231 as target cells, and the expression of HER-2 had been verified (Supplementary Figure S7). IL-7 and IL-7+IL-21 cytokines cocktails were excluded from killing experiments, due to its insufficiency in generating applicable T cells. HER-2 CAR-T cells and SK-BR-3 were co-cultured at different effector-to-target ratios. Cell culture supernatant was collected and CAM release was measured to evaluate the anti-tumor effect of HER-2 CAR-T cells under each culture condition. Meanwhile, concentration of indicated effector cytokines released into the supernatant was measured after 24 h incubation. Results showed that the tumor-specific killing ability of HER-2 CAR-T cells was enhanced by addition of IL-21 at all E:T ratios (Figure 6A). Similar to the results of killing assay, the expression of effector cytokines (IFN-γ and granzyme B) increased by addition of IL-21 in the cytokine cocktails (Figures 6B,C). Similar results were obtained using SW480 as target cells (Supplementary Figure S8). As for the HER-2-negative MDA-MB-231 cells, the CAR-T cells had no significant killing effect and secretion of effector cytokines, as compared with the mock T cells (Figures 6D–F).

### IL-15+IL-21 is the Most Efficient Cytokine Combo for CAR-T Cell Expansion and Effector Function

**FIGURE 7:**
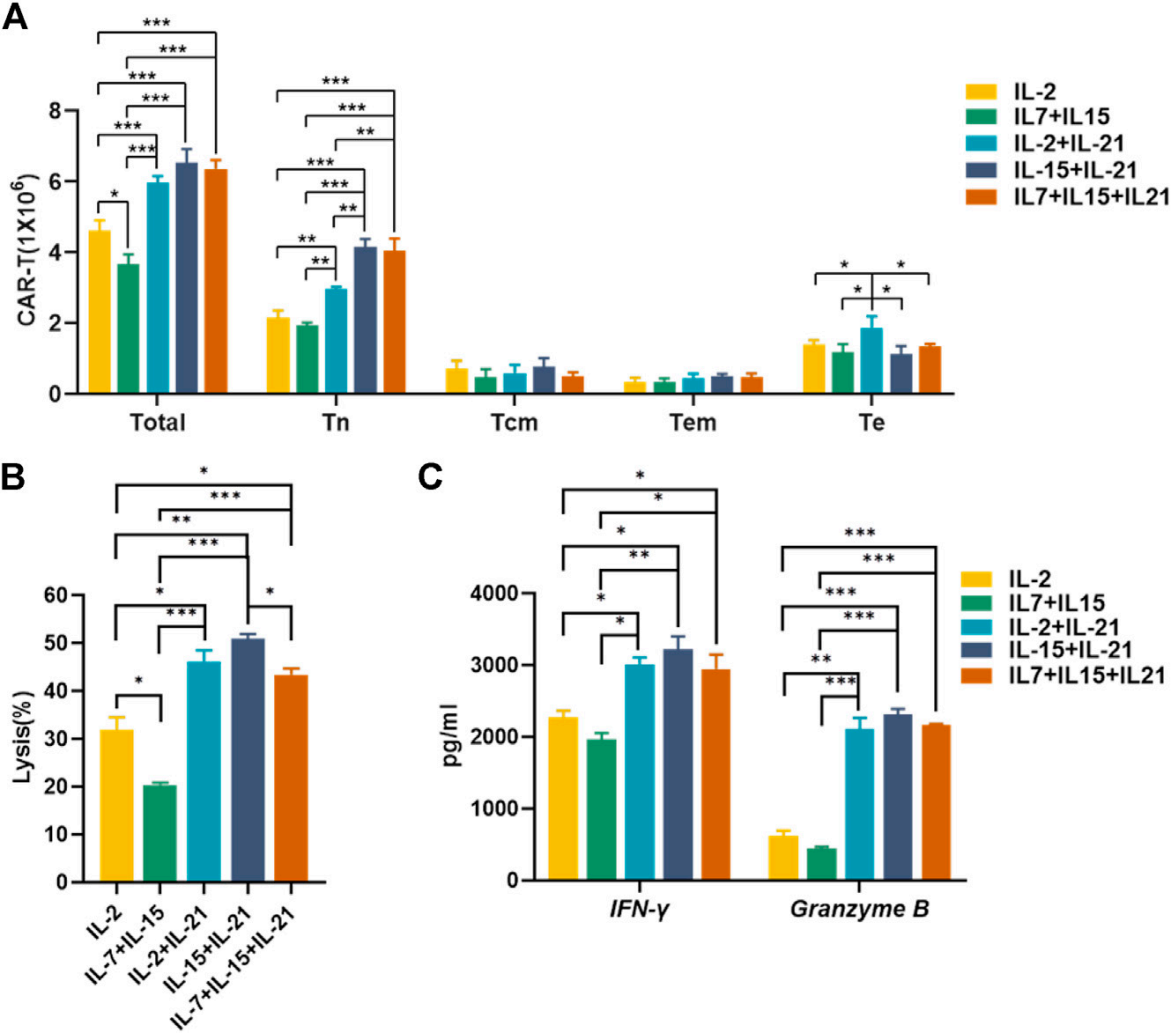
IL-15+IL-21 is the optimal cytokine combination for the CAR-T cell preparation *in vitro*. **(A)** The absolute numbers of gated total CAR-T cells and different CAR-T subtypes were assessed on day 12 after transfection. **(B)** Cytotoxicity of HER-2-specific CAR-T cultured under different cytokine conditions was evaluated by Calcein AM release assay after co-culture with SK-BR-3 for 6 h at an effector to target ratio of 2:1. **(C)** Supernatant was collected after 24 h co-culture of HER-2 CAR-T cells with SK-BR-3 at 2:1 effector-to-target ratio and assayed for IFN-γ and granzyme B release by ELISA. Each experiment was performed in triplicate. Mean values were calculated for each group. Error bars represent ±SEM. Statistical significance was calculated using Student *t* test, *p < 0.05, **p < 0.01, ***p < 0.001.

To identify the optimal cytokine combinations for CAR-T preparation, we compared the absolute numbers and effector function of CAR-T cells cultured with the selected cytokine combinations. The absolute number of total CAR-T cells was significantly higher in the IL-2+IL-21, IL-15+IL-21 and IL-7+IL-15 + IL21 combos, as compared to the IL-2 and IL-7+IL-15 combos. But there was no significant difference in total amount of CAR-T cells among the IL-2+IL-21, IL-15+IL-21 and IL-7+IL-15 + IL21 combos. IL-15+IL-21 and IL-7+IL-15 + IL21 cocktails exhibited a largest number of Tn^+^ CAR-T cells, and IL-2+IL-21 had the largest number of Te^+^ CAR-T cells among the five groups (Figure 7A). Therefore, the IL-15+IL-21 and IL-7+IL-15+IL-21 treatment combos were considered to be associated with the best amplification of CAR-T cells, as well as the largest number of Tn^+^ CAR-T cells.

Due to sufficient cytotoxic activity of HER-2 CAR-T cells against HER-2-positive target cells at high effector: target ratios, we chose low effector: target ratios to test the killing efficiency of CAR-T cells cultured with selected cytokine cocktails. The IL-15+IL-21 showed the strongest cytotoxic activity (Figure 7B). While the IL-2+IL-21, IL-15+IL-21 and IL-7+IL-15 + IL21 combos exhibited similar effector cytokines secretion, (Figure 7C). For the best amplification of CAR-T cells and the strongest cytotoxic activity, we concluded that IL-15+IL-21 was the optimal cytokine combo for the CAR-T preparation.

## Discussion

In this study, we found that the addition of IL-21 in the commonly used cytokine cocktails enhanced lentiviral transfection efficiency of T cells, which was independent of the enrichment and expansion of less differentiated CAR-T cells by addition of IL-21. Then, we found that IL-21 inhibited the expression of IFN-γ at the moment of T cell transfection, which partially explained the increased transfection efficiency. We also confirmed that IL-21 enhanced CAR-T cell cytotoxicity (Batra et al., 2020). Together, our results suggest that large number of high-quality CAR-T cells can be obtained with simple addition of IL-21 in the CAR-T preparation.

Our finding that transfection efficiency of T cells was enhanced by addition of IL-21 had not been matched with previous researches, which suggest that IL-21 has been investigated as a therapeutic modality in a number of viral infections, such as HIV (Adoro et al., 2015; Shen et al., 2020). We speculated that the contradictory may be resulted from the different T cell activation state at the moment of infection. To verify the speculation, we detected the expression of IFN-γ, which was assessed as a measure of T cell activation and effector function (Li and Yee, 2008). We found that IFN-γ expression of T cells was significantly downregulated at the moment of T cell transfection by addition of IL-21. Conversely, we observed T cell IFN-γ expression was elevated by IL-21 during the very early stage of T cell activation. This finding suggests that the effect of IL-21 on the functional properties of T cells may vary during different time periods. The mechanism of IL-21 in regulating T cell IFN-γ expression remains unknown. And the mere detection of IFN-γ expression is not sufficient, other important markers (e.g., CD107a and CD137) should also be detected to investigate the functional role of IL-21 in T cells.

Despite a considerable amount of research regarding the cytokine conditions used for the CAR-T cell preparation (Zeng et al., 2005; Kagoya et al., 2018; Mata and Gottschalk, 2019), due to the various preparation methods, and experimental conditions, it is difficult to determine the optimal cytokine combinations for the CAR-T cell preparation by comparing experimental results in the different literature. We compared the absolute numbers and effector function of CAR-T cells cultured with different cytokine cocktails. The outstanding performance in proliferation and cytotoxicity of CAR-T cells cultured with IL-15+IL-21 led to our conclusion that IL-15+IL-21 was the optimal cytokine combinations for the CAR-T cell preparation. Such advancement of IL-15+IL-21 has been independently confirmed with the potential regulation from TCF-1 (Sutherland et al., 2013). Insufficient expansion of CAR-T cells cultured with IL-7 or IL-7+IL-21 combos might result from the reduced IL-7R expression on naive T cells upon activation (Park et al., 2004). And insufficient expansion of CAR-T cells cultured with IL-7+IL-21 combo indirectly suggested that addition of IL-21 alone in the CAR-T preparation may have minimal effects on T cell proliferation. Because the levels of cytokine receptors expression vary during different stages of T cell activation (Schluns and Lefrançois, 2003), precise supplementation of cytokine cocktails based on expression levels of cytokine receptors during different stages of T cell activation may be taken into consideration in a follow-up experiment. Considering transfection manipulation might introduce some effects on T cell expansion and subtypes, we compared the proliferation and phenotypes of activated T cells generated without transfection. The data obtained was consistent with the results above (data not shown).

Currently, there are many CAR-T cell preparation methods in clinical trials. Our CAR-T cell preparation is merely one of many methods for the preparation of CAR-T cells. Considering the diversity of CAR-T cell preparation methods, our study does have some limitations. But there exist similarities in many CAR-T cell preparation approaches. On the one hand, although different groups are using different reagents to activate T cells, the mechanism of activation of T cells is the same via anti-CD3/anti-CD28 stimulation. On the other hand, nowadays, most teams are still using lentivirus transfection in CAR-T cell clinical trials. Furthermore, our transfection operation steps are simple, and multiple transfection replicates are not required. We do not use polybrene and retronectin for lentivirus packaging and T cell transfection, which showed some degree of cytotoxicity to the T cells. Thus, the present study may act as a reference for fundamental research and some clinical trials in the future. CAR-T cell products from individuals are costly and the use of IL-21 in CAR-T clinical trials certainly further increases the economic costs. But the advantages of IL-21 in CAR-T cell preparation would be likely to promote the widespread use of IL-21 in T cell-related experiments, which in turn may reduce its cost. Of course, our study has a number of limitations that should also be noted. Further and more comprehensive studies are required to confirm these findings. And additional in vivo experiments are needed to verify the in vitro results and examine the side effects of CAR-T cells.

In summary, we found for the first time that IL-21 improved lentivirus transfection efficiency of T cells through reduction of IFN-γ expression at the moment of T cell transfection. On the other hand, we confirmed that IL-21 enhanced the enrichment and expansion of less differentiated CAR-T cells. Further, IL-21 augmented the CAR-T cell cytotoxicity and secretion of IFN-γ and granzymes B. Results from this study suggest that the addition of IL-21 in the cytokine cocktails is a potential approach for optimizing CAR-T cell preparation.

## Data Availability

The original contributions presented in the study are included in the article/Supplementary Material, further inquiries can be directed to the corresponding author.
