# Supplementary figures and images for: IL-21 Optimizes the CAR-T Cell Preparation Through Improving Lentivirus Mediated Transfection Efficiency of T Cells and Enhancing CAR-T Cell Cytotoxic Activities
Source: Front Mol Biosci. 2021 Jun 4;8:675179. doi: 10.3389/fmolb.2021.675179 (PMC8220804; doi:10.3389/fmolb.2021.675179)

## Slide 1
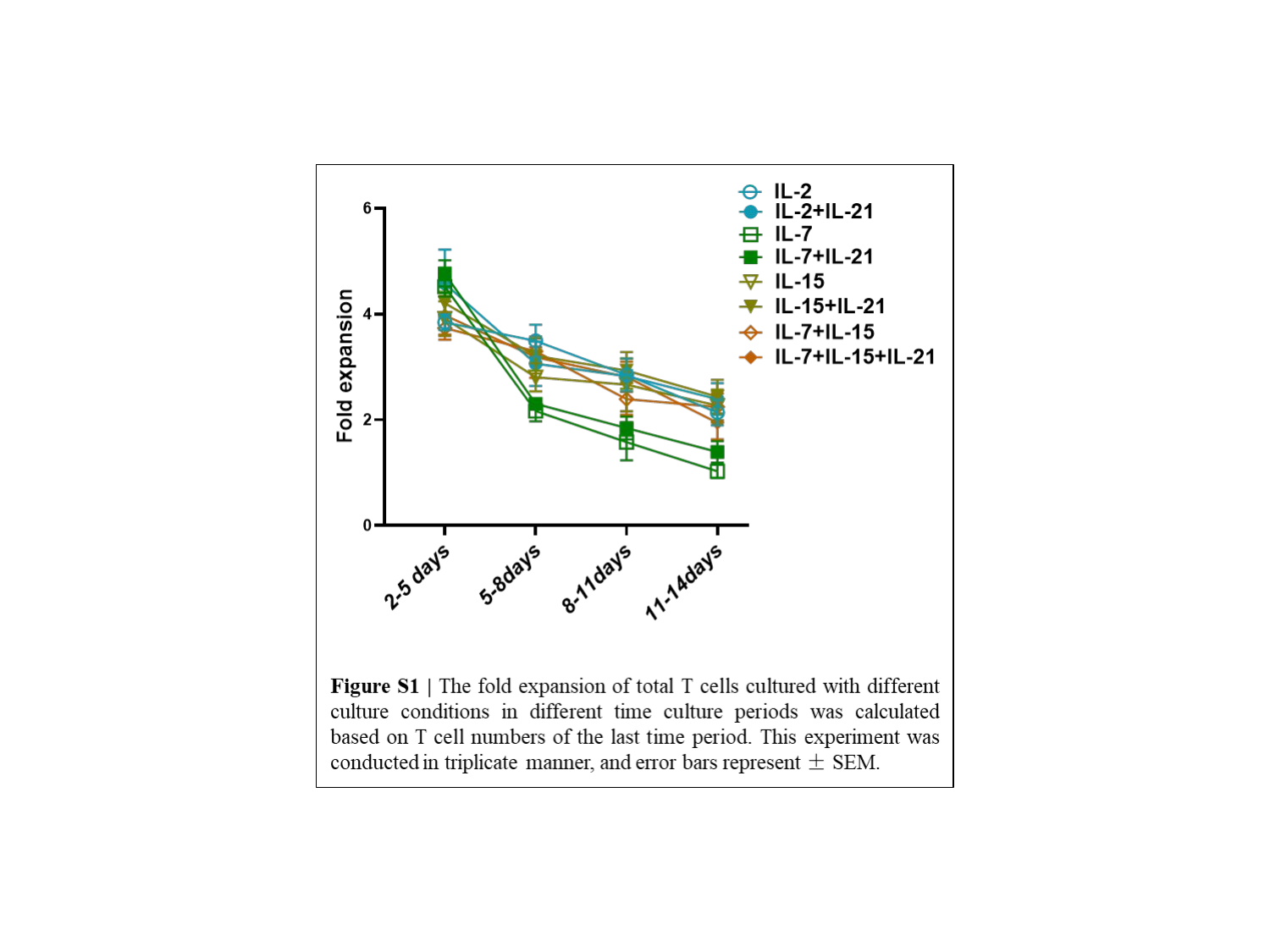

## Slide 2
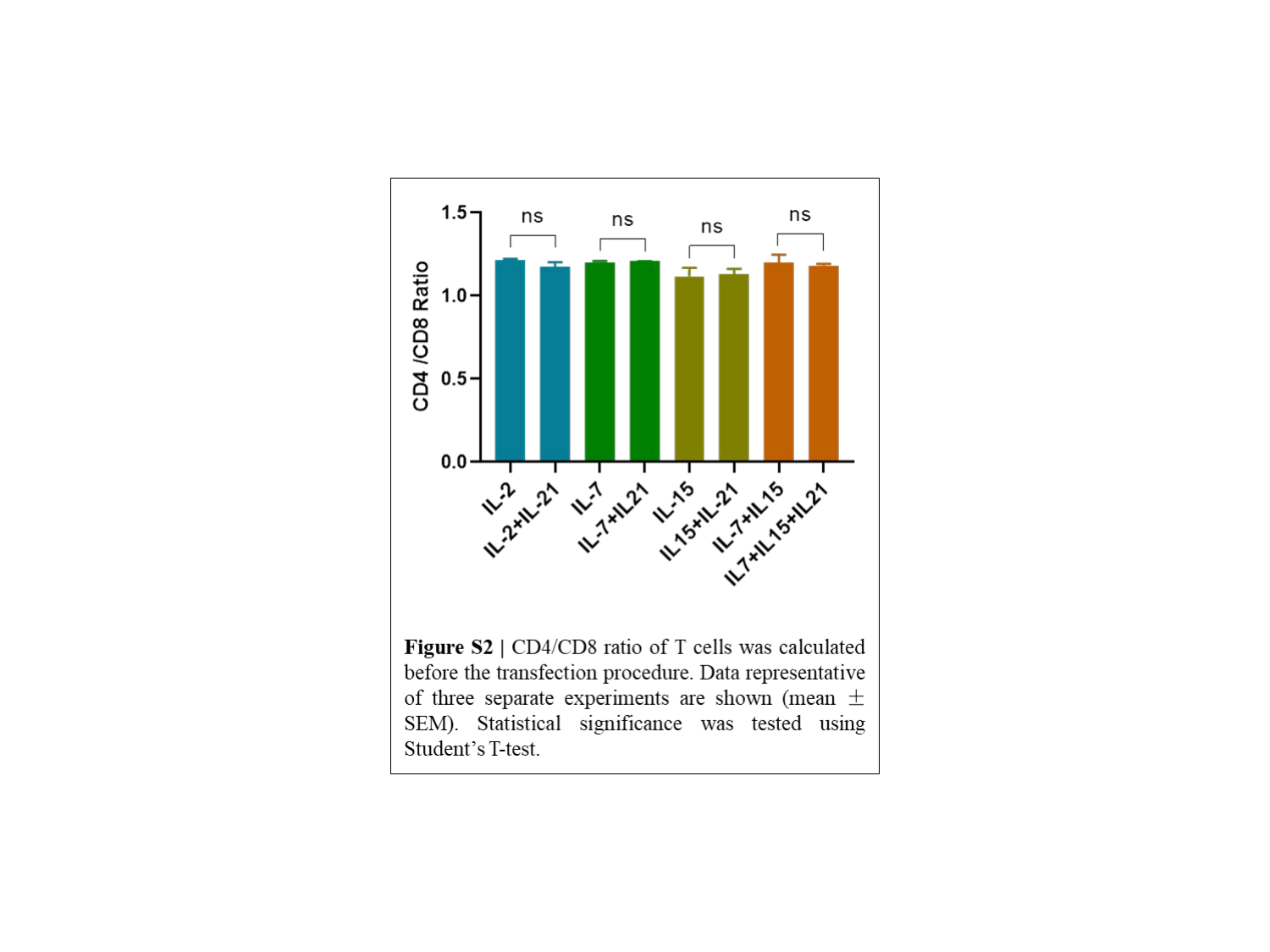

## Slide 3
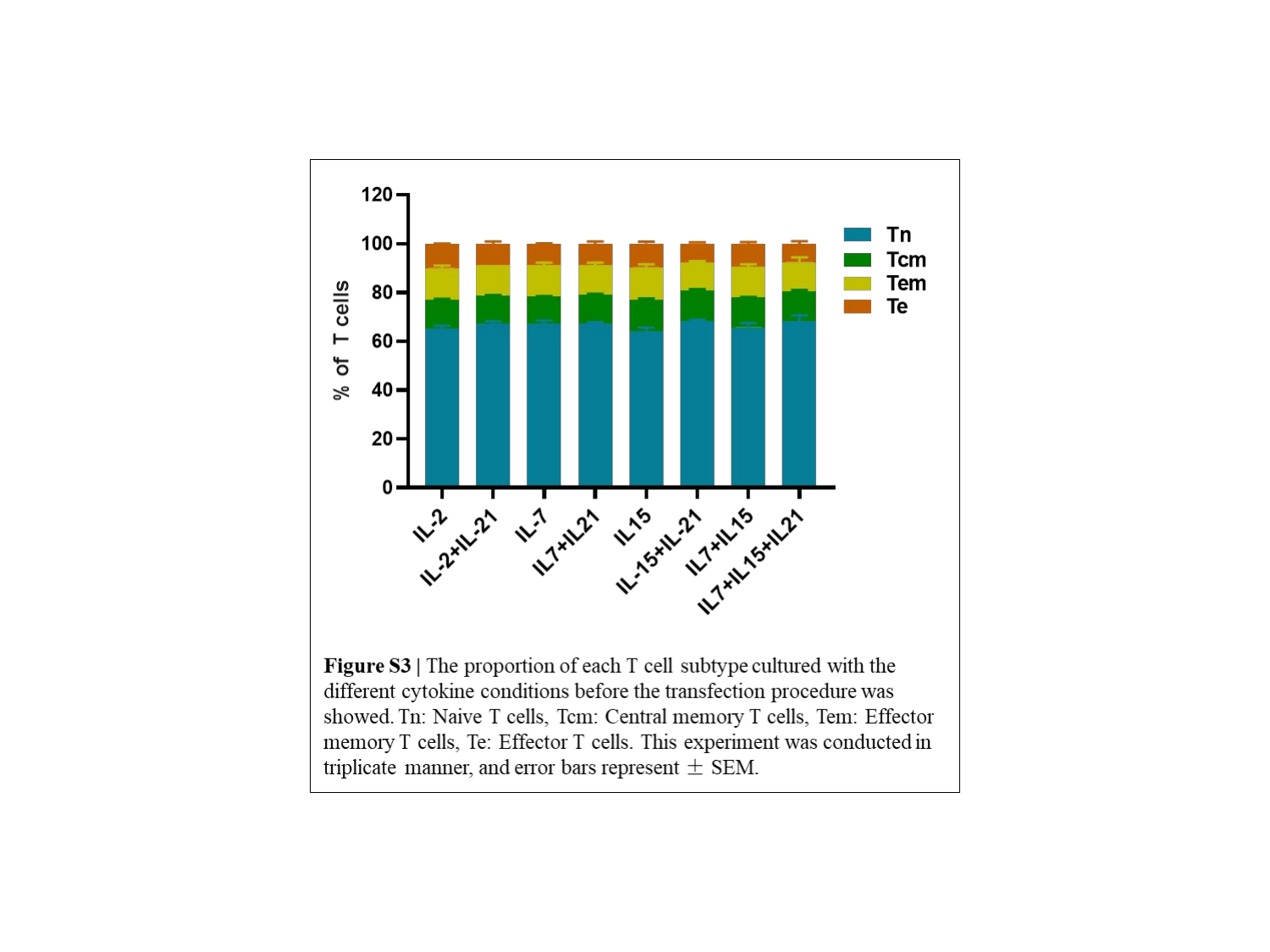

## Slide 4
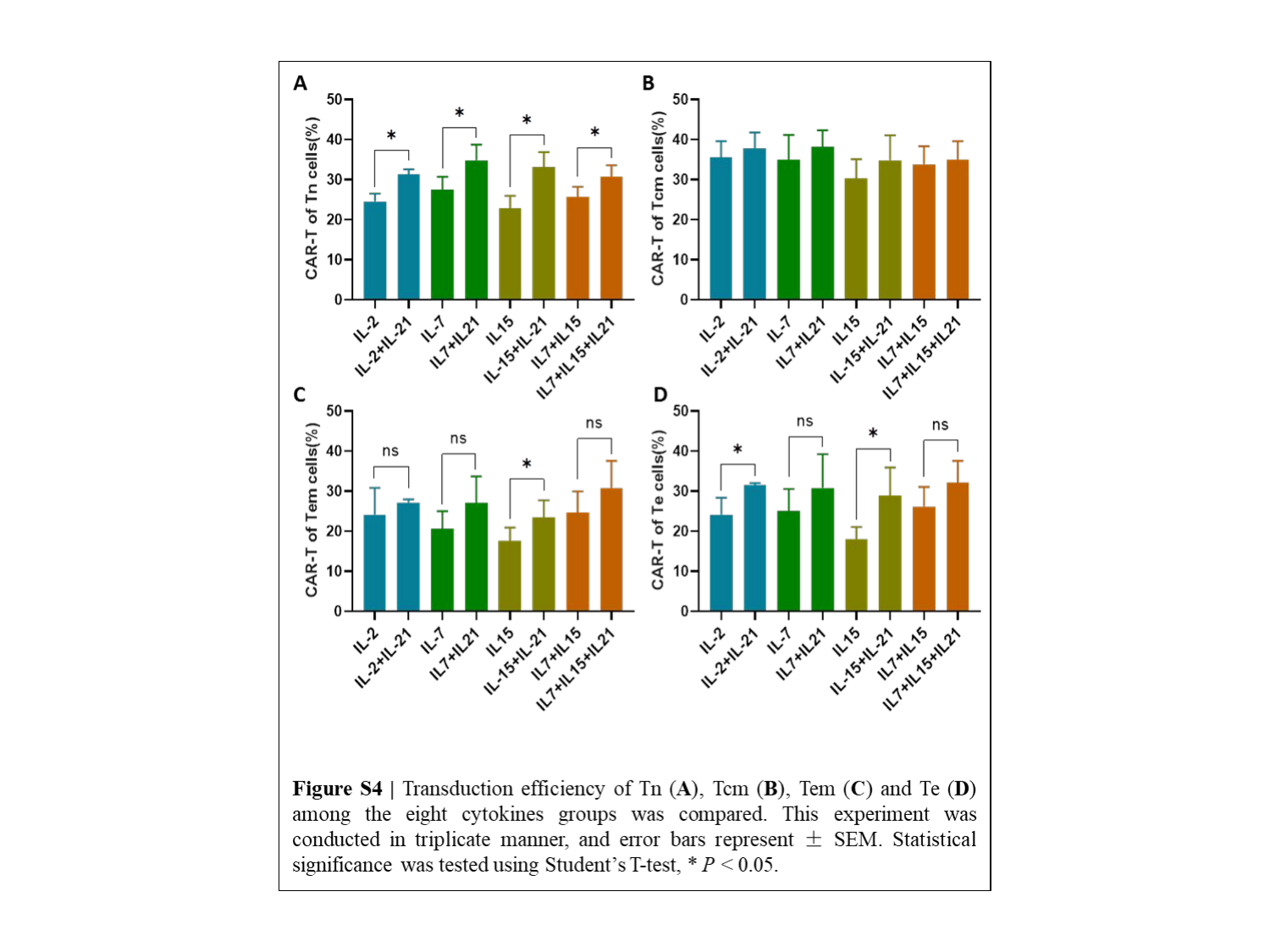

## Slide 5
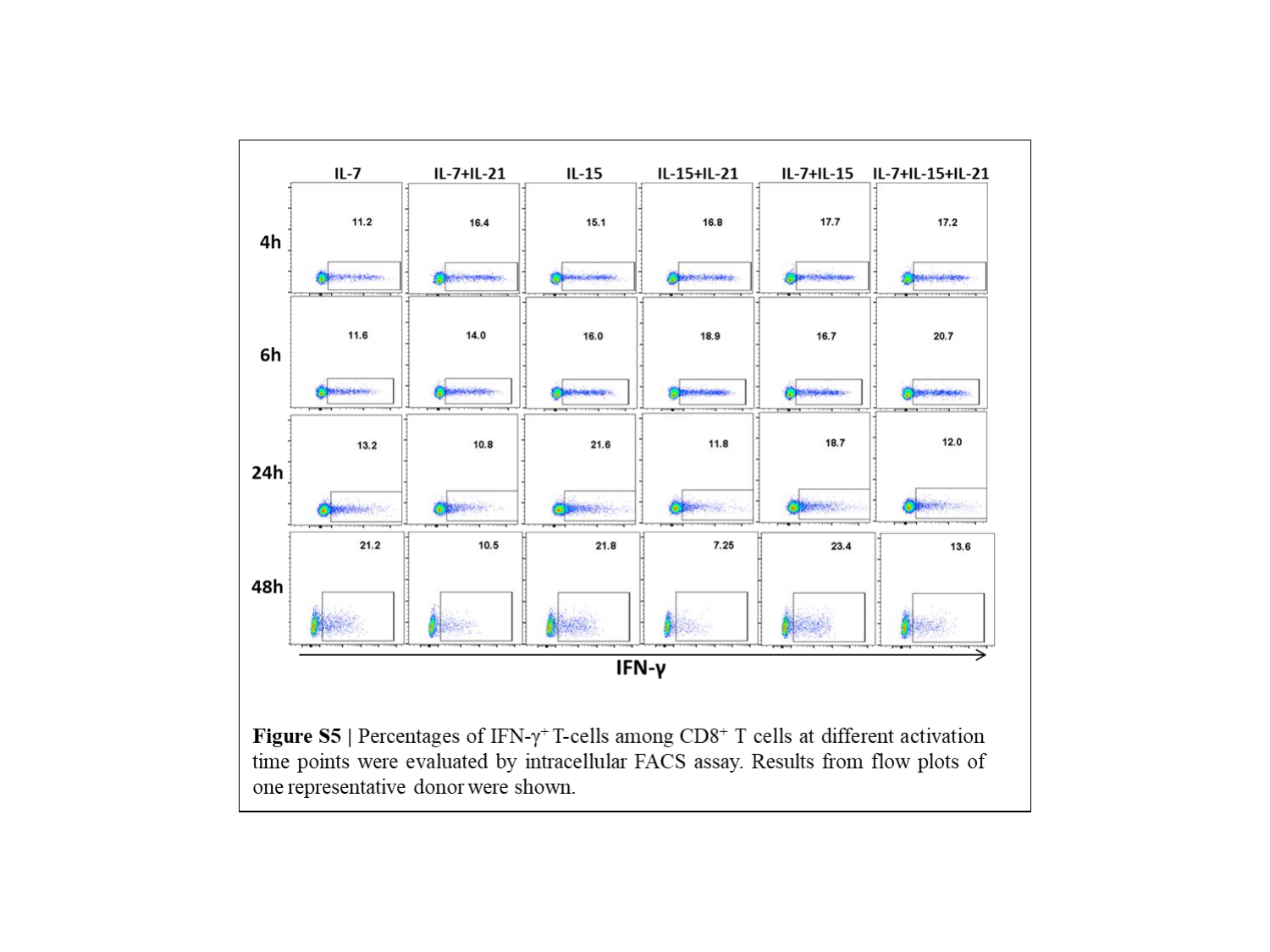

## Slide 6
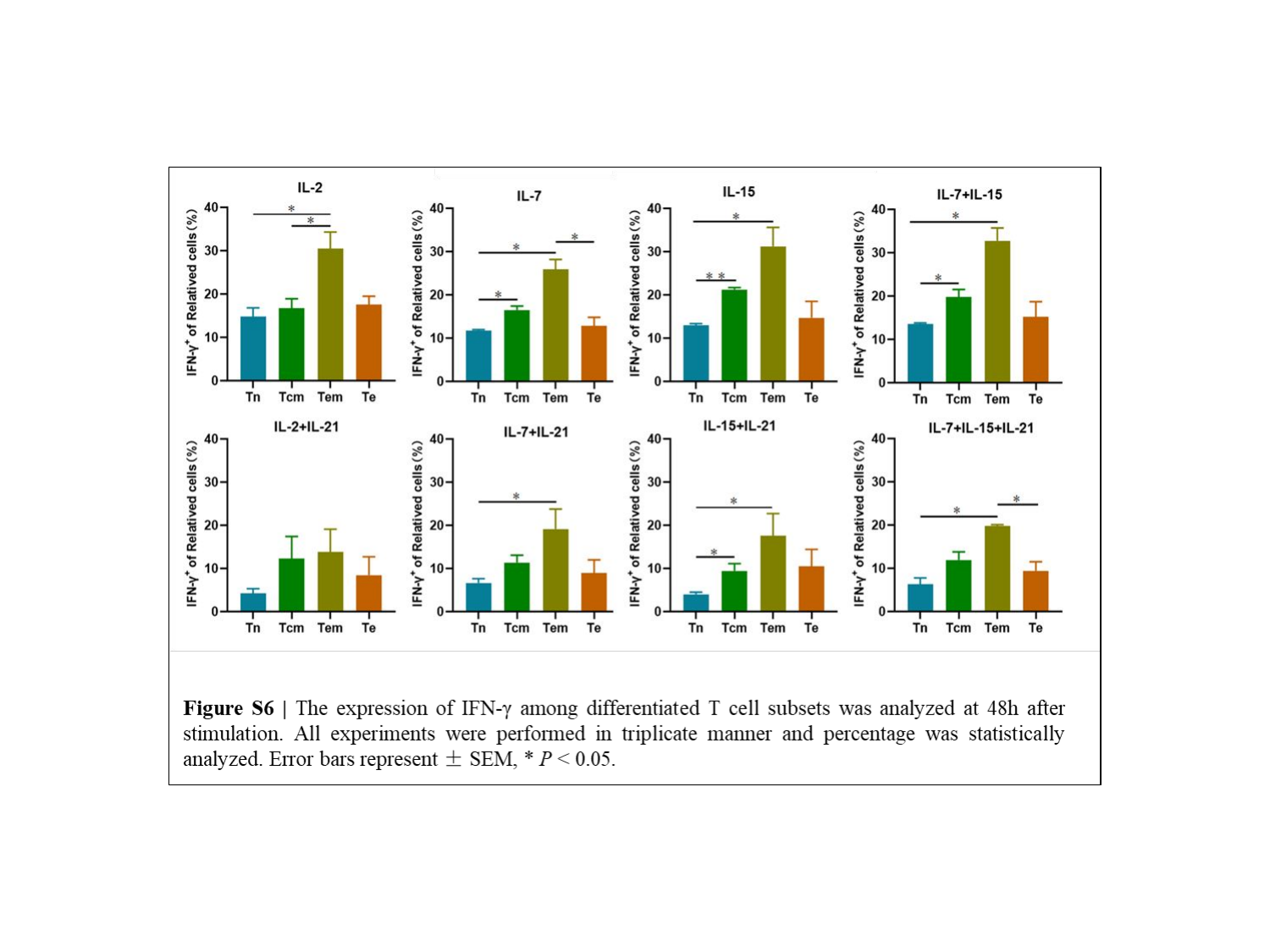

## Slide 7
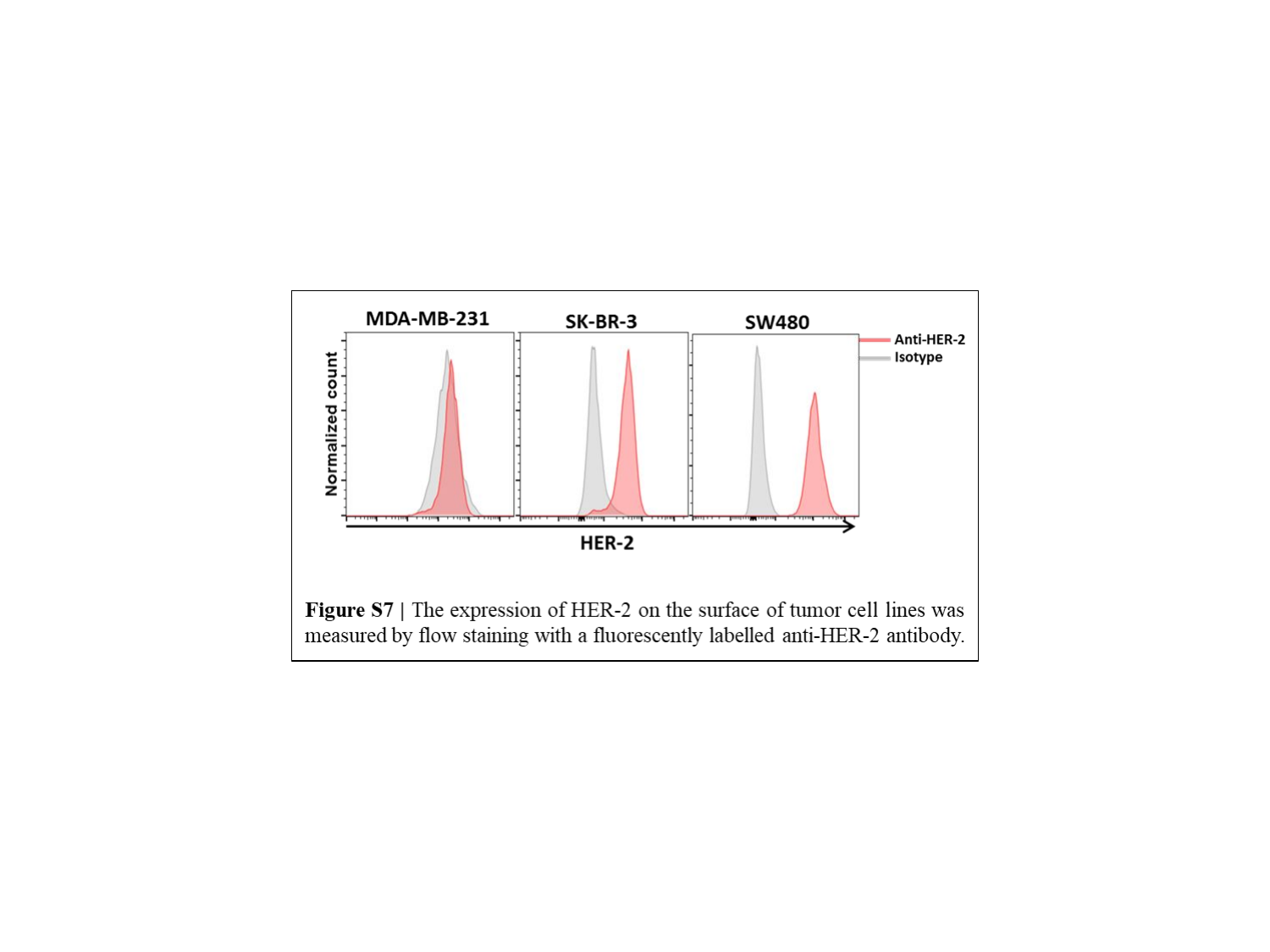

## Slide 8
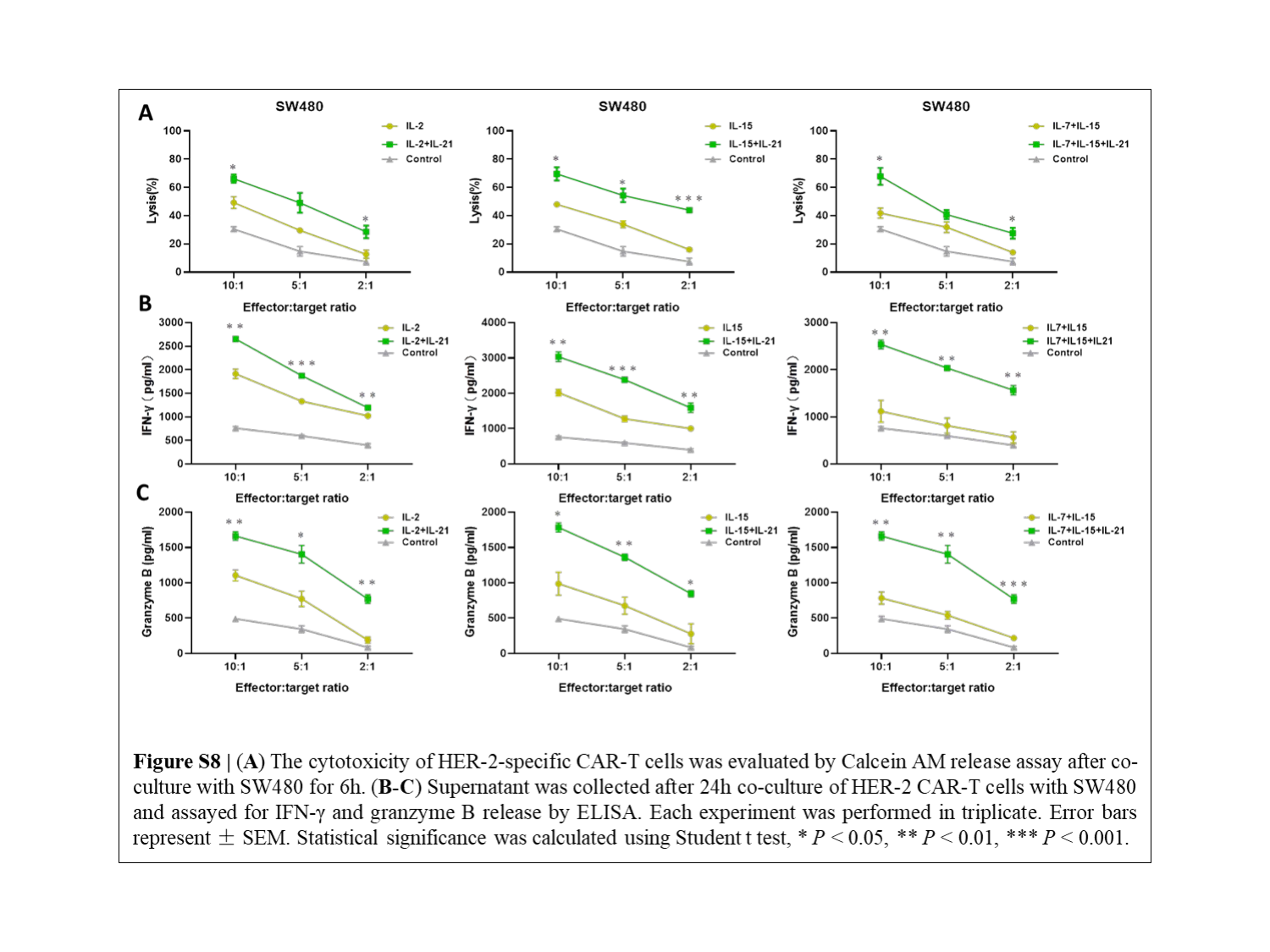

Supplement: Supplementary file 1 [file Presentation1.PPTX]
